# Dialectical behavior therapy (DBT) in an assertive community treatment structure (ACT): testing integrated care borderline (ICB) in a randomized controlled trial (RECOVER)

**DOI:** 10.1186/s40479-024-00261-4

**Published:** 2024-08-14

**Authors:** Andreas Schindler, H. F. Warkentin, J. Bierbrodt, H. König, A. Konnopka, A. Pepic, J. Peth, M. Lambert, J. Gallinat, A. Karow, H.-H. König, M. Härter, H. Schulz, A. Rohenkohl, K. Krog, S. V. Biedermann, I. Schäfer

**Affiliations:** 1 Center for Psychosocial Medicine, Department of Psychiatry and Psychotherapy, University Medical Center Hamburg-Eppendorf, Hamburg, Germany; 2 Center for Psychosocial Medicine, Department of Health Economics and Health Services Research, University Medical Center Hamburg-Eppendorf, Hamburg, Germany; 3 Center for Experimental Medicine, Department of Medical Biometry and Epidemiology, University Medical Center Hamburg-Eppendorf, Hamburg, Germany; 4 Center for Psychosocial Medicine, Department of Medical Psychology, University Medical Center Hamburg-Eppendorf, Hamburg, Germany

**Keywords:** Integrated care borderline (ICB), Borderline personality disorder (BPD), Dialectical behavior therapy (DBT), Assertive community treatment (ACT)

## Abstract

**Background:**

Though Dialectical Behavior Therapy (DBT) and other treatment models for individuals with Borderline Personality Disorder (BPD) have shown to be efficient in inpatient and outpatient settings, there is a general shortage of these treatments. In Germany, most resources are spent on inpatient treatments and unspecific crisis interventions, while it is difficult to implement the necessary team structures in an outpatient setting. This study is testing an alternative approach focussing on outpatient treatment: Integrated Care Borderline (ICB) provides DBT for persons with severe BPD within the structures of an Assertive Community Treatment (ACT). ICB is team-based, integrating psychiatric and social support as well as crisis interventions into a DBT-strategy.

**Methods:**

ICB was compared to TAU in a prospective, randomized controlled trial. This study is part of RECOVER, a comprehensive stepped care approach in Germany, which enrolled a total of 891 participants. 146 persons were diagnosed with BPD as main diagnosis. Of these, 100 were allocated to the highest level of severe mental illness (SMI) and randomly assigned to either ICB (*n* = 50) or TAU (*n* = 50). Data were collected at baseline and 12 months later. The main outcomes were psychosocial functioning (GAF), severity of BPD (BSL-23) and other mental symptoms (BSI, PHQ-9, GAD-7, self-harm), employment status (VILI), as well as hospital days and associated costs.

**Results:**

Data show a significant increase of psychosocial functioning and a significant decrease of BPD and other psychiatric symptoms in both groups (*r* = .28 – .64), without any significant differences between the groups. The proportion of self-harming persons decreased in both groups without statistical significance. Patients were significantly more likely to be employed after a year of treatment in ICB (*p* = .001), but not in the TAU group (*p* = .454). Analyses showed a significant difference between the groups (*p* = .032). Moreover, psychiatric hospital days were significantly reduced in ICB (-89%, *p* < .001, *r* = .61), but not in TAU (-41%, *p* = .276, *r* = .15), resulting in a significant difference between the groups (*p* = .016) and in lower annual hospital costs in ICB (5,546€ vs. 10,726€, -48%, *p* = .011) compared to TAU.

**Conclusion:**

Our results replicate earlier studies, showing that DBT can be efficient in outpatient settings. Furthermore, they indicate additional effects on employment and hospital days. The ICB-approach seems to offer a viable framework for multiprofessional outpatient DBT-teams. Future research will have to test whether the additional effects are brought about by the additional features of ICB compared to standard outpatient DBT.

**Trial registration:**

Registration number with ClinicalTrials.gov (NCT03459664), RECOVER.

## Introduction

Borderline personality disorder (BPD) is a complex disorder, causing tremendous distress in patients and relatives, and posing a challenge to any treatment system. BPD has a point prevalence of 0.8 to 2.0% [1] and a lifetime prevalence of 5.9% [2]. About one third of these patients meet the criteria of a severe mental illness (SMI) [3]. SMI is understood as a disorder resulting in serious functional impairment [4], usually operationalized as a GAF-score (General Assessment of Functioning [5]) below 50 and a duration of at least 2 years [6]. Treatment approaches for patients with severe BPD have to be able to deal with repetitive self-harming and suicidal crises. Given the long-term course and the complexity of the disorder with psychological, social, and somatic issues, the development of treatment teams [7] and networks as well as models of integrated care (IC) [8] and stepped care have been recommended [9, 10].

However, treatment structures for these patients usually fall short of these requirements [11]. Though research has shown four different psychotherapeutic treatment approaches to be effective [12] in outpatient as well as in inpatient settings [13–16], there is a general quantitative and qualitative shortage of these treatments in all 22 countries examined [11]. Ratios of treatment-seeking patients with BPD to mental health professionals ranged from 4:1 to 192:1 and regarding professionals certified in providing evidence-based care, ratios ranged from 49:1 to 148215:1 depending on the country [11]. This lack of effective treatment leads to an increased demand for emergency treatment and to increasing costs [16–18] as patients are repeatedly admitted to psychiatric hospitals in acute crisis situations. In the US, BPD-patients accounted for 43% of all adult psychiatric admissions for suicide risk [16]. Though often inevitable in acute situations, crisis admissions seem to have adverse effects [19], leading to a three times higher risk to be readmitted to a psychiatric hospital within the same year [16]. In Germany, unspecific crisis interventions account for 70% of the inpatient treatment costs of BPD, while only 30% are spent on evidence-based inpatient psychotherapy. Outpatient therapies represent just one-tenth of inpatient treatment costs [18]. BPD-patients account for 15% of all inpatient psychiatric treatment cases [20]. With an average of 70 hospital days p.a., they are responsible for 25% of the total costs of these treatments [20]. Internationally, these figures reflect inadequate treatment systems. On the one hand the scarce supply leads to long waiting lists and difficulties to get an access to treatment, on the other hand resources are spent on expensive and inefficient treatments [10, 11].

Several authors have called for stepped-care-approaches to redistribute resources according to the severity of the disorder [9–11]. Preliminary data suggest that treatment duration and hospital days can be reduced for the less severely ill [21, 22]. However, the most evident solution would be an improvement of evidence-based outpatient treatment teams, which are equally effective as inpatient treatments, but less expensive [21]. In Germany, the most frequently offered treatment is Dialectical Behavior Therapy (DBT) [23]. Though DBT was originally developed as an outpatient treatment for suicidal BPD-patients, in Germany it is regularely offered as a three-months inpatient treatment. Although there is a growing number of DBT-therapists working in an outpatient setting, it is difficult to organize the necessary teams to provide adherent DBT. DBT-networks, which have shown to be effective [15], are only available in a few cities. The basic DBT-notion of a team treating a group of patients is applied in hospital settings, but not in the German outpatient health care system that mainly relies on individual therapists treating individual patients.

Looking for models to implement team structures in outpatient treatment we would like to propose the structure of an Assertive Community Treatment (ACT). ACT models have shown to be an efficient approach in SMI-samples of persons with psychosis and bipolar disorders. ACT-models are mainly working in an outpatient setting and they are able to reduce hospital days and costs. At the same time, they lead to a better social functioning, better housing, and employment status in this group [24].

ACT might have several advantages for the treatment of BPD: (1) ACT is a team-based structure that can provide the framework for a DBT-team. (2) ACT teams are multiprofessional, consisting of psychotherapists, psychiatrists and social workers. This allows social issues and medication to be integrated into a DBT-strategy. (3) If these teams are based in or closely connected to a psychiatric hospital, they can manage crisis interventions in outpatient and inpatient settings.

To date only two uncontrolled pilot studies provide preliminary evidence that DBT within a comprehensive structure of ACT might improve quality of life and occupational functioning and reduce hospital days [25, 26]. The current study is part of RECOVER, a comprehensive stepped care approach recently tested in Germany [27]. It is the first RCT testing a model of integrated care for severely ill BPD-patients. Integrated Care Boderline (ICB) is providing DBT in the structures of an Assertive Community Treatment (ACT).

## Integrated care for BPD-patients (ICB)

In order to improve the treatment situation of severely ill BPD-patients, the ICB-model [28, 29] has been developed based on the structures of ACT-models for psychotic patients, especially the well-evaluated “Hamburg model” of IC-psychosis [30]. The model is financed by flat-rate case fees. It includes case management, psychotherapeutic, psychiatric, and socio-therapeutic treatment, as well as crisis intervention in an outpatient or inpatient setting. The core of ICB is a multiprofessional outpatient team of psychotherapists, psychiatrists, and social workers. The team is located in a psychiatric hospital, using all of its facilities if needed.

**Fig. 1:**
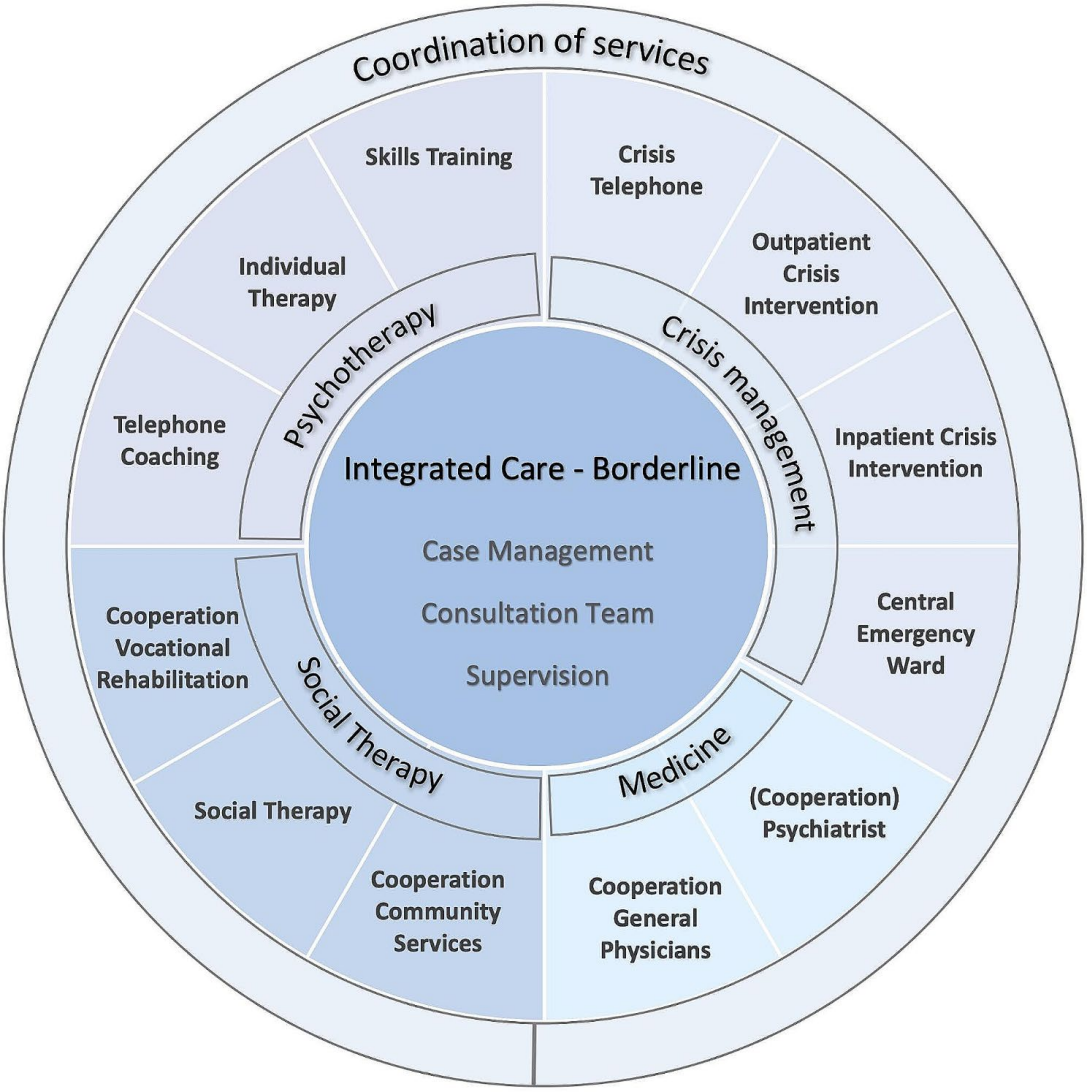
Structure of the integrated care-borderline (IC-B) concept

Since BPD-patients cannot be successfully integrated into ACT-models for psychotic patients [31], these structures need to be adapted with a specific, evidence-based approach for the psychotherapeutic treatment of BPD. To date, DBT is the approach with the broadest evidence. It offers a clear framework and is suited for severely ill BPD-patients. DBT requires a team of therapists treating a group of patients, reflecting on the treatment and deciding treatment steps together in a weekly consultation team. It combines individual and group therapy with individualized crisis intervention plans and telephone coaching. ICB follows DBT routines [23] and additionally integrates psychiatric and socio-therapeutic treatments. And though it mainly works in an outpatient setting, it offers the possibility of inpatient crisis intervention and home treatment. The advantage of integrating psychiatrists, social workers and crisis intervention is that these issues can be handled within an DBT strategy. This means that the team does not have to rely on external providers who may not always follow a DBT strategy. Its main goals are to promote functional behavior and reduce dysfunctional behavior, to develop a better management of crises, and to reduce inpatient treatment. The model is described in detail in several papers and a book [28, 29]. To ensure evidence-based and DBT-adherent treatment, all team members in ICB received a 96 h DBT-training and were supervised monthly by approved DBT-experts from the German “DBT-Dachverband”. A consultation team was run on a weekly basis. Figure 1 shows the elements of the ICB model.

## Methods

### Study design

This study compares a sample of BPD-patients receiving ICB-treatment to a randomized sample of BPD-patients receiving treatment as usual (TAU). The two main hypotheses were that persons being treated in ICB (1) show a better patient-related outcome, including psychosocial functioning, symptom severity and social integration, and (2) reduced hospital days and hospital costs compared to TAU within a one-year treatment period. Data were collected in the RECOVER study [27, 32]. The study was approved by the local ethics committee. RECOVER is a parallel-group RCT testing a comprehensive, stepped-care-model of mental health treatment. It classified participants into four levels of severity (see next paragraph). ICB represented the treatment for the most severely ill persons with BPD within the RECOVER model. Participants were recruited from 2018 to 2020 in the city of Hamburg, Germany. Managed care network partners were contacted to identify potentially eligible patients. Participants were consecutively invited to participate by members of the research team and received compensation for the increased time spent during follow-up visits. All research members were trained by independent research institutes in conducting the interviews and using the rating scales.

### Definition and classification of severity levels

As a model of stepped care, RECOVER includes the entire spectrum of mental disorders and defines four levels of severity: (1) mild, (2) moderate, (3) severe, and (4) persistent SMI with complex care needs. The definition of severity is based on specific diagnoses (here: BPD) and a combined criterion of disease severity (Clinical Global Impression Scale, CGI-S [33]) and psychosocial functioning level (Global Assessment of Functioning Scale, GAF [5]). Participants in the present study needed to have a CGI-score of 5 (markedly ill) to 7 (extremely ill) and a GAF-score below 50 (severe impairment in several areas).

### Participants

**Fig. 2:**
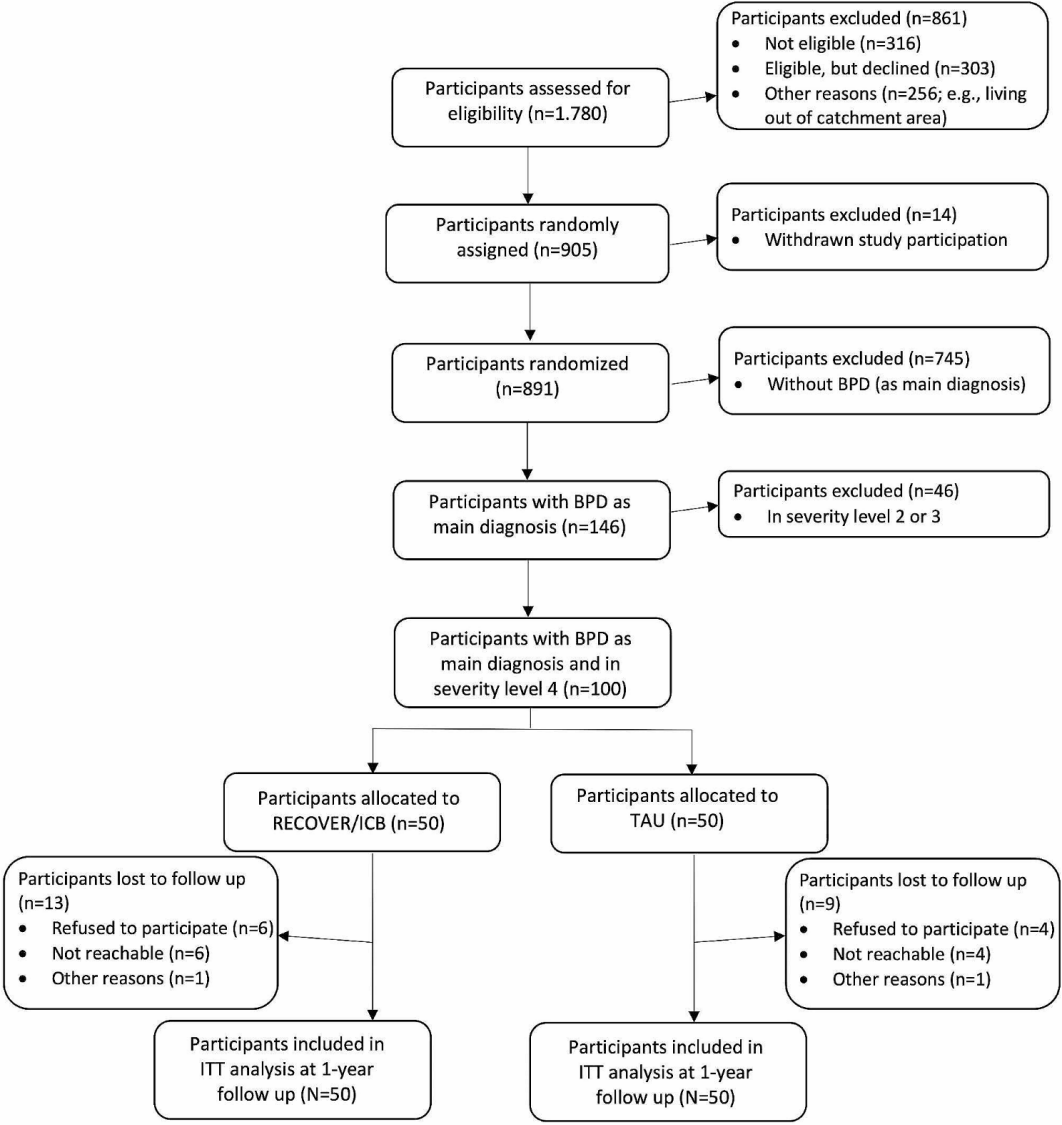
CONSORT flow chart of participants

Between 2018 and 2020, RECOVER screened a total of 1780 patients of whom 905 consented and were randomly assigned either to the intervention or to the control group (454 to RECOVER and 451 to TAU). This paper focuses exclusively on persons in severity level 4 which were treated in ICB as a treatment arm of RECOVER for severily ill BPD-patients. Persons with less severe symptoms (level 1–3) were treated in other, less intensive RECOVER treatment options, that are described and analyzed in other publications [32]. Inclusion criteria were based on the target group of ICB treatment which addresses adult patients who meet the criteria of SMI / RECOVER level 4 and have a main diagnosis of BPD (ICD-10: F60.31) without excluding other comorbid psychiatric disorders. Patients had to live in an 8 km catchment area of the hospital, so that they could reach the clinic in a crisis and so that home treatment is possible if required. Patients had to be insured by one of the participating health insurance companies. Exclusion criteria in the entire RECOVER study were organic mental disorders (F0), severe mental retardation (F72, F73), and insufficient language skills. Of 905 randomized patients in the RECOVER study, 100 patients met these criteria. Of those, 50 were randomized in ICB and 50 in TAU. 22 patients withdrew or had missing primary outcomes at the 12-months follow up (Post) (*n* = 9 (18%) in ICB, *n* = 13 (26%) in TAU group. As an intent-to-treat (ITT) sample, the data of all patients randomized at baseline (Pre) were imputed and used in the statistical analyses (See Fig. 2).

### Randomization and masking

After a comprehensive baseline assessment and informed consent patients were assigned to one of the four levels of severity. Then, each patient was randomized in a 1:1 ratio from a list of random numbers previously generated for each severity level using the ralloc method (STATA-SE 14). Research members involved in randomization were excluded from data collection, those collecting follow-up data were blinded regarding the group assignment, just as those responsible for data analysis were blinded regarding the treatment assignment until finalization of a statistical analysis plan.

### Experimental and control intervention

In the RECOVER model, severe mentally ill patients are treated by diagnosis-specific IC-teams. Please see introduction for a description of the ICB model. Patients in the control group were treated as usual (TAU) within the standard care of the local catchment area. TAU included the use of all the hospital’s services, offering DBT in a three-months inpatient (or alternatively: day-care) setting, an outpatient team providing diagnostics and outpatient skills-groups in collaboration with external therapists and psychiatrists. Additionally, the catchment area is relatively well equipped with specialists in general medicine, psychiatry and psychotherapy, with psychological psychotherapists in private practice, and with facilities for the rehabilitation of mental illness. Contrary to ICB, TAU-patients did not get an overall treatment plan or case management.

### Measures

Data were collected at baseline (Pre) and twelve months later (Post). All instruments are well established standard measures with good psychometric properites. Psychosocial functioning was assessed with the Global Assessment of Functioning Scale (GAF) [5]. Psychiatric symptoms were measured using self-report questionnaires: BPD symptoms were assessed using Borderline Symptom List (BSL-23) [34]. Here, internal consistency is Cronbach’s α = .94 for the overall score. Brief Symptom Inventory (BSI) [35] was used for general psychiatric symptoms. In this study, Cronbach’s α is .96 for the Global Severity Index BSI GSI. Patient Health Questionnaire (PHQ-9) [36] was used for depressive symptoms. Cronbach’s α here is .84. Anxiety symptoms were measured with Generalized Anxiety Disorder (GAD-7) [37]. In our data, Cronbach’s α amounts to .84. Self-harm was assessed using a dichotomous item (self-harmed in the last months/did not self-harm in the last months). As a measure of social integration, the employment status was used which was assessed by the VILI [38]. Patients were categorized as “employed” (working, in education) or “not employed” (sick, unemployed, retired). Service utilization was operationalized as days spend in day-care or inpatient treatment in the last year each twelve months before Pre and the twelve months between Pre and Post.

Health care utilization was assessed using the Questionnaire to Assess Medical and Non-Medical Resource Utilization in Mental Disorders (FIMPsy) [39] and the Questionnaire on Utilization of Medical and Non-Medical Services in the Elderly (FIMA) [40]. Standardized unit costs were assigned to the categories of care [41, 42]. The total societal costs were separated into health and social care costs and productivity costs due to being “not employed”. Hospital costs (inpatient and day-care) were analyzed separately from outpatient physician/psychologist costs and other health and social care costs.

### Statistical analysis

RECOVER sample size planning was based on a power calculation to detect statistically significant differences between groups of a small to moderate effect size (Cohen’s f of 0.175, i.e., Cohen’s d of 0.35) at 12 months (Post) in the primary outcome measure of psychosocial functioning. This resulted in a total of 890 study participants (445 in each group) [27, 32]. Out of these, data from 100 patients with severe BPD were used for this paper.

In-group-differences in psychosocial functioning, psychiatric symptoms, and psychiatric hospital days were analyzed using Wilcoxon signed-rank tests and paired t-tests depending on the distribution of the variables. Between-group differences were calculated by linear models (ANCOVA). Differences in the respective values between Post and Pre were used as dependent variables, group (ICB vs. TAU) as fixed factor, and the respective baseline variable as covariate to minimize variance. Changes in the proportion of self-harming and (un-)employed individuals between Pre and Post were analyzed using McNemar’s test. Differences in these changes between the ICB and TAU groups were calculated using a logistic regression model, where the self-harm/employment status at Post was used as dependent variable, the self-harm/employment status at baseline and the group variable as independent variable.

Costs were analyzed using adjusted generalized linear models with Poisson distribution and log-link function. These models were adjusted for age, sex, respective baseline costs, somatic illnesses, illness severity, The total societal costs and productivity costs were additionally adjusted for employment. Data were checked against violations of the assumptions of the analyses [43]. Missing values in baseline and follow-up data were imputed using the EM algorithm (expectation maximization). The two-sided type I error was set to α = 5%. All analyses are performed in an explorative manner without adjustment for multiplicity. Analyses were performed using IBM SPSS Statistics 25.

## Results

### Sample characteristics

Baseline sociodemographic and clinical characteristics are given in Table 1. Chi-squared and Mann-Whitney-U-tests showed no statistically significant differences in these characteristics between the groups. About three quarters of the total sample were female, single and unemployed at baseline. The mean GAF score was well below the cutoff of SMI indicating severe symptoms or severe impairment in functioning. Almost a quarter did not receive any treatment at baseline. 95% of the total sample had at least one comorbid mental disorder with major depressive disorder (75%), PTSD (39%), and substance dependence (36%) being the most common.

**Table 1:** Sample characteristics at baseline of ICB intervention and TAU control group

	ICB (*n* = 50)	TAU (*n* = 50)	Total (*N* = 100)	Difference	
Age (years, mean (SD))	31.7 (10.2)	32.4 (11.8)	32.0 (11.0)	*U* = 1,248.50	*p* = .992
Sex (female, n (%))	39 (78.0%)	36 (72.0%)	75 (75.0%)	*χ*^2^=0.48	*p* = .645
Marital status (single, n (%))	31 (62.0%)	39 (78.0%)	70 (70.0%)	*χ*^2^=3.05	*p* = .126
Educational level (at least 12 years, n (%))	22 (44.0%)	24 (48.0%)	46 (46.0%)	*χ*^2^=0.16	*p* = .841
Vocational status (unemployed, N (%))	40 (80.0%)	36 (72.0%)	76 (76.0%)	*χ*^2^=0.88	*p.*=483
Comorbid disorders					
Comorbid axis I mental disorder (yes, n (%))	44 (88.0%)	47 (94.0%)	91 (91.0%)	*χ*^2^=1.10	*p* = .487
Comorbid axis II mental disorder (yes, n (%))	13 (26.0%)	14 (28.0%)	27 (27.0%)	*χ*^2^=0.05	*p* > .999
Comorbid somatic disorder (yes, n (%))	32 (64.0%)	28 (56.0%)	60 (60.0%)	*χ*^2^=0.67	*p* = .541
Treatment at baseline					
Inpatient (hospital) (yes, n (%))	10 (20.0%)	12 (24.0%)	22 (22.0%)	*χ*^2^=0.23	*p* = .810
Day clinic (hospital) (yes, n (%))	6 (12.0%)	1 (2.0%)	7 (7.0%)	*χ*^2^=3.84	*p* = .112
Outpatient (yes, n (%))	19 (38.0%)	28 (56.0%)	47 (47.0%)	*χ*^2^=3.25	*p* = .109
No treatment (yes, n (%))	15 (30.0%)	9 (18.0%)	24 (24.0%)	*χ*^2^=1.97	*p* = .241
GAF score (mean (SD))	43.6 (5.7)	42.2 (6.1)	42.9 (5.9)	*U* = 1,072.00	*p* = .218
CGI score (mean (SD))	5.4 (0.6)	5.4 (0.5)	5.4 (0.6)	*U* = 1,285.00	*p* = .781
Inpatient days (hospital) in the last 12 months (mean (SD))	39.0 (69.5)	27.2 (49.2)	33.1 (60.2)	*U* = 1,188.00	*p* = .640
Day-care days (hospital) in the last 12 months (mean (SD))	24.4 (44.8)	13.5 (36.1)	18.9 (40.9)	*U* = 1,035.00	*p* = .051
Suicidal attempt in the past (yes, n (%))	26 (52.0%)	27 (54.0%)	53 (53.0%)	*χ*^2^=0.04	*p* > .999
Self-harm in the past (yes, n (%))	40 (80.0%)	41 (82.0%)	81 (81.0%)	*χ*^2^=0.00	*p* > .999
Age of disorder onset (mean (SD))	14.0 (6.7)	15.3 (5.8)	14.6 (6.3)	*U* = 1,447.50	*p* = .173
Age of initial psychiatric contact (mean (SD))	22.0 (12.3)	23.4 (13.4)	22.7(12.8)	*U* = 1,310.00	*p* = .679

### Time and group effects

#### Psychosocial functioning

Psychosocial functioning (GAF) improved significantly in both groups between Pre and Post. Contrary to expectations, the ICB group did not show a larger increase than TAU (see Table 2).

#### Psychiatric symptoms

Differences in borderline symptoms (BSL-23), general psychiatric symptoms (BSI), depressive (PHQ-9), and anxiety symptoms (GAD-7), between Pre and Post as well as between the groups were calculated (see Table 2). We could see a significant symptom reduction over time in both groups and in all variables examined. Again, data did not show any significant differences between groups. For the dichotomous variable of self-harm, the rate of self-harming persons changed from 56 to 42% (-14%) in the ICB group, whilst the rate of self-harming persons in the TAU group reduced from 52 to 40% (-12%). This reduction was statistically non-significant for both groups. There were no relevant differences between the groups.

**Table 2:** In- and between-group differences in psychosocial functioning and psychiatric symptoms of ICB and TAU groups over one year

In group differences after 12 months								Between group differences after 12 months				
**Variable**	**group**	**Baseline (Pre; M (SD))**	**One year (Post; M (SD))**	**Observed difference (M (SD))**		**p**	**Effect size**	**Adjusted mean (95% CI)** ^2^			**Adjusted difference** ^2^	**p**
Psychosocial functioning (GAF)	ICB^1^	43.64 (5.66)	49.93 (9.62)	6.29 (7.93)		**< 0.001**	0.64	6.41 (4.00, 8.83)			0.07 (-3.36, 3.49)	0.970
	TAU^1^	42.22 (6.11)	48.70 (9.96)	6.48 (9.18)		**< 0.001**	0.61	6.35 (3.94, 8.77)				
Borderline symptoms (BSL-23)	ICB	2.10 (0.82)	1.81 (1.01)	-0.29 (0.94)		**0.036**	0.31	-0.31 (-0.53, -0.08)			0.17 (-0.15, 0.49)	0.489
	TAU	2.24 (0.92)	1.74 (1.02)	-0.49 (0.72)		**< 0.001**	0.68	-0.47 (-0.70, -0.25)				
Overall psychiatric symptoms (BSI)	ICB	1.98 (0.72)	1.66 (0.88)	-0.32 (0.84)		**0.009**	0.38	-0.33 (-0.52, -0.13)			0.14 (-0.15, 0.42)	0.336
	TAU	1.98 (0.73)	1.52 (0.84)	-0.46 (0.63)		**< 0.001**	0.73	-0.46 (-0.66, -0.26)				
Depressive symptoms (PHQ-9)	ICB	17.12 (5.27)	14.93 (6.97)	-2.19 (5.49)		**0.007**	0.4	-2.25 (-3.74, -0.76)			1.24 (-0.86, 3. 43)	0.244
	TAU	17.48 (5.94)	13.93 (5.95)	-3.55 (5.68)		**< 0.001**	0.63	-3.49 (-4.98, -2.01)				
Anxiety symptoms (GAD-7)	ICB	14.15 (4.09)	11.96 (5.41)	-2.19 (4.65)		**0.002**	0.43	-2.12 (-3.41, -0.83)			-0.43 (-2.25, 1.39)	0.638
	TAU	13.81 (4.98)	12.20 (5.07)	-1.61 (5.25)		**0.045**	0.28	-1.69 (-2.97, -0.40)				
**Variable**	**group**	**Baseline (Pre; %)**	**One year (Post; %)**	**Observed difference (%)**	**p**		**B**	**SE**	**Wald**	**p**	**Odds ratio**	**95% CI**
Self-harm (SH)	ICB	28 (56.0)	21 (42.0)	7 (14.0)	0.265	SH at pre^4^	-0.12	0.42	0.09	0.037	2.39	1.06–5.40
	TAU	26 (52.0)	20 (40.0)	6 (12.0)	0.377	Group	-0.87	0.42	4.36	0.769	1.13	0.50–2.56
						Constant	1.14	0.93	1.51	0.219	3.14	
Employment status (ES; employed)	ICB	10 (20.0)	28 (56.0)	18 (36.0)	**0.001**	ES at pre^4^	-0.94	0.42	4.94	0.109	0.45	0.17–1.19
	TAU	14 (28.0)	18 (36.0)	4 (8.0)	0.454	group	0.7	0.5	1.98	**0.032**	2.47	1.08–5.64
						Constant	0.35	0.84	0.17	**0.679**	1.41	

**Notes**: ^1^ ICB/TAU treatment group, each *n* = 50; ^2^ Adjusted for the respective baseline variable; ^4^ Model results for binary logistic regressions; SH: *X*^*2*^(2) = 4.50, *p* = .105; Nagelkerke pseudo *R*^*2*^ = 0.06; ES: *X*^*2*^(2) = 6.59, *p* = .037; Nagelkerke pseudo *R*^*2*^ = 0.09

#### Social integration

Regarding the employment status, the number of employed persons more than doubled from 20 to 56% (+ 26%; *p* = .001) in the ICB group while in the TAU group it increased from 28 to 36% (+ 8%; *p* = .276). The logistic regression showed the group affiliation to be a significant predictor of the outcome.

#### Hospital days

For both groups, the number of psychiatric hospital days was reduced in the year between Pre and Post compared to the year before Pre, with the ICB group showing a significantly larger difference (-89%, *p* < .001) compared to the TAU group (-41%, *p* = .276). The difference between the groups is -16.62 (95% CI -30.31 to -3.12; *p* = .016) days, meaning a difference of more than two weeks in day-care or inpatient treatment per year between the groups (see Tables 3 and 4).

**Table 3:** In- and between-group differences in service utilization of ICB intervention and TAU control groups over one year

In group differences after 12 months							Between group differences after 12 months		
**Variable**	**group**	**Baseline (Pre; M (SD))**	**One year (Post; M (SD))**	**Observed difference (M (SD))**	**p**	**Effect size**	**Adjusted mean (95% CI)** ^3^	**Adjusted difference** ^3^	**p**
Psychiatric	ICB^2^	63.41 (76.16)	7.00 (16.82)	-56.42 (80.39)	**<0.001**	0.61	-44.94 (-54.43, -35,45)	-16.62 (-30.13, -3.12)	**0.016**
hospital days^1^	TAU^2^	40.72 (69.22)	23.88 (44.21)	-16.84 (81.29)	0.276	0.15	-28.32 (-37.81, -18.82)		

**Notes**:^1^ in the last twelve months before Pre/Post; ^2^ ICB/TAU treatment group, each *n* = 50; ^3^ Adjusted for baseline psychiatric hospital days.

### Societal and health care costs

Table 4 shows the adjusted cost differences between ICB and TAU. The total annual societal costs were lower in the ICB than in the TAU group (18,369€ vs. 23,759€, -23%, *p* = .094), though this difference failed to reach statistical significance. Differences resulted from different health and social care costs, which were significantly lower in the ICB group (-4,840€, -25%, *p* = .050), as costs for incapacity of work did not differ significantly between the groups (-368€, -8%, *p* = .855). The reduction of service utilization results in a halving of the day-care and inpatient hospital costs in ICB compared to TAU (5,546€ vs. 10,726€, -48%, *p* = .011). Outpatient costs for physicians and psychologists were non-significantly higher in the ICB group (+ 626, + 24%, *p* = .089).

**Table 4:** Adjusted total costs and between-group differences of ICB intervention and TAU control group in adjusted costs of care over one year

Annual cost of care category ^1–3*^	ICB group (*n* = 50)	TAU group (*n* = 50)	ICB group vs. TAU group (*N* = 100)	
	Annual costs (in €, M (SE))	Annual costs (*n* = 50)	Annual costs difference (in €, %)	*p*
Total societal costs	18,369 (1,864)	23,759 (2,575)	-5,390 (-23%)	0.094
Total health and social care costs	14,272 (1,373)	19,122 (2,082)	-4,840 (-25%)	**0.050**
Total hospital costs	5,546 (973)	10,726 (1,788)	-5,180 (-48%)	**0.011**
Inpatient hospital costs	3,923 (775)	8,248 (1,745)	-4,325 (-52%)	**0.024**
Day-care hospital costs	1,498 (365)	2,671 (693)	-1,173 (-44%)	0.121
Outpatient costs for physicians / psychologists	3,271 (254)	2,645 (271)	+ 626 (+ 24%)	0.089
Other health and social care costs	5,252 (706)	5,797 (728)	-545 (-9%)	0.593
Productivity costs due to sick leave	4,142 (1,281)	4,510 (1,272)	-368 (-8%)	0.855

**Notes**: ^1^ Adjusting costs for: age, sex, respective baseline costs, somatic illnesses, illness severity, calculation with generalized linear models with Poisson distribution and log-link function. The total societal costs and productivity costs were additionally adjusted for employment. ^2^ Total health and social care costs: inpatient and day-care stays, outpatient physician contacts, outpatient healthcare providers, medication, nursing/care, informal care, rehab, counseling & support, vocational integration, crisis interventions, web-based services, peer support. ^3^ Other health and social care costs: outpatient healthcare providers, medication, nursing/care, informal care, rehab, counseling & support, vocational integration, crisis interventions, web-based services, peer support

*Prices in € and from the year 2019

M = mean, SE = standard error

## Discussion

The present study is the first randomized-controlled trial testing a model of Integrated Care for persons with severe BPD. Sample characteristics underpin that severe BPD is a complex mental illness associated with various psychiatric, somatic, and social impairments: the majority of the sample was unemployed, not engaged in a romantic relationship, and suffered from at least one other psychiatric and somatic illness. The majority had already self-harmed, and more than half of the sample had attempted to commit suicide in the past. In Germany, these severily ill BPD-patients are usually treated in inpatient settings. This study tested an approach that mainly worked in an outpatient setting using a DBT-approach within an ACT-structure. Its main results are:

Persons in ICB-treatment showed an equivalent improvement in psychosocial functioning and symptom reduction as patients in standard care. Both groups showed higher GAF scores as well as a reduction in borderline, general psychiatric, depressive, and anxiety symptoms after one year of treatment. Though we expected ICB-patients to show stronger improvements in these clinical measures, data do not confirm any substantial differences between ICB and TAU. This indicates that ICB as well as TAU, including DBT in different settings in a university medical center, are efficient treatments. These results are in tune with earlier studies showing that DBT is an efficient treatment for severily ill BPD-patients in outpatient as well as inpatient settings [13, 18, 20, 44]. However, there were two significant differences between groups: ICB was associated with fewer hospital days compared to TAU. While participants in the TAU group spent 24 days in in psychiatric hospitals, this figure was reduced to seven days in ICB-patients. This reduction of hospital days of course led to a significant reduction of hospital costs in ICB compared to TAU (€5,546 vs. €10,726). With its intensive outpatient treatment approach, ICB had slightly, though non-significantly higher outpatient treatment costs than TAU, reflecting the reallocation of resources from inpatient to outpatient treatment. This shift is thought to reduce total annual treatment costs, which is visible in a non-significant tendency in our data (€18,369 ICB vs. €23,759 TAU). More research is needed to test these possible cost reductions.

With regard to social integration persons in the ICB group were more likely to be employed after a year of treatment than persons in the TAU group. Though this did not immediately reduce societal costs for incapacity of work, it emphasizes the usefulness of integrating socio-therapeutic services into ICB treatment. Additionally, this effect might be a consequence of the reduced hospital days: patients can work while in outpatient treatment, but cannot when in hospital. These results are in tune with findings from ACT-studies in samples of persons with psychosis and bipolar disorder, where ACT typically led to a reduction of hospital days and a better social integration [24].

### Limitations

Though RECOVER included a huge clinical sample, sample size in this sub-study focusing on severe BPD was too small. Several potentially meaningful results failed to reach significance. Future studies should include larger samples of BPD-patients. This will need multi-center-designs and longer recruitment times.

A further limitation is the comparison of ICB to TAU in a university medical center, including a variety of treatments and settings. Though this reflects the reality of the treatment system in Germany and highlights ecological validity, future studies should compare ICB to other specific treatment models like inpatient or outpatient DBT. These studies should focus on the question of whether the additional features of ICB have an additional effect compared to standard DBT. The positive effect on employment in this study suggests that the integration of social work is helpful. However, the data do not allow any conclusions about the effect of integrating psychiatrists into the team. The reduction of hospital days could indicate a more efficient crisis intervention strategy. As we did not assess whether the patients were admitted for planned treatments or for crisis interventions, we cannot draw this conclusion. The course of BPD usually lasts for years if not decades. We only analyzed outcomes after one year of treatment. Future studies should cover longer treatment and follow-up periods.

## Conclusion

In conclusion, our results indicate a benefit of combining DBT and ACT. They replicate the well established finding of DBT as an efficient treatment for severe BPD. They show that an ACT-structure seems to be a viable framework for an outpatient DBT-team, that additionally helps to improve employment and to reduce hospital days.

## Data Availability

The datasets used and/or analyzed during the current study are available from the corresponding author on reasonable request.

## Abbreviations

ACT: Assertive Community Treatment
ANCOVA: Analysis Of Covariance
BPD: Borderline Personality Disorder
BSI: Brief Symptom Inventory
BSL-23: Borderline Symptom List-23
CGI: Clinical Global Impression Scale
CI: Confidence Interval
DBT: Dialectical Behavior Therapy
ES: Employment Status
GAD-7: Generalized Anxiety Disorder 7
GAF: General Assessment of Functioning
IC: Integrated Care
ICB: Integrated Care Borderline
ITT: Intent-To-Treat
M: Mean
PHQ-9: Patient Health Questionnaire 9
RCT: Randomized Controlled Trial
SD: Standard Deviation
SE: Standard Error
SH: Self Harm
SMI: Severe Mental Illness/Severely Mentally Ill
TAU: Treatment As Usual
